# Toward Quantitative End-Group Fidelity in the Synthesis of High Molecular Weight Polysarcosine

**DOI:** 10.1021/acsmacrolett.5c00165

**Published:** 2025-04-15

**Authors:** Zlata Nagorna, Matthias Barz, Joachim F. R. Van Guyse

**Affiliations:** † Leiden Academic Centre for Drug Research (LACDR), 4496 Leiden University, Einsteinweg 55, 2333, CC, Leiden, The Netherlands

## Abstract

Polymers applied in pharmaceutical applications need to meet stringent quality standards to ensure reproducibility of product properties, such as efficacy and safety of therapeutics. End-group fidelity is a crucial quality feature that ensures functional integrity, reproducible synthesis, and robust therapeutic performance. The contemporary production of poly­(ethylene glycol) (PEG) exemplifies this requirement, which has consolidated its position as a gold standard in pharmaceutical applications. However, modest to severe immune responses toward PEG in patients generate the need for alternative polymers in the development of pharmaceuticals or cosmetics. Among such alternatives, polysarcosine (pSar) displays PEG-like stealth properties in vivo while displaying improved immunogenicity and toxicity profiles, generating the need for heterotelechelic pSar polymers of the highest end-group integrity. Here, we compared current synthetic methods for the controlled synthesis of pSar over a broad molecular weight range and assessed the end-group fidelity by ion exchange chromatography. Subsequent isolation allowed the identification of impurities via mass spectrometry, thus yielding mechanistic insights into the *N*-substituted *N*-carboxyanhydride ring-opening polymerization (ROP). Our results reveal a nuanced role of organocatalysts in the ROP, highlighting opportunities for better catalysts. Finally, this work showcases a scalable purification method to obtain high molecular weight pSar with quantitative end-group fidelity.

## Graphical abstract

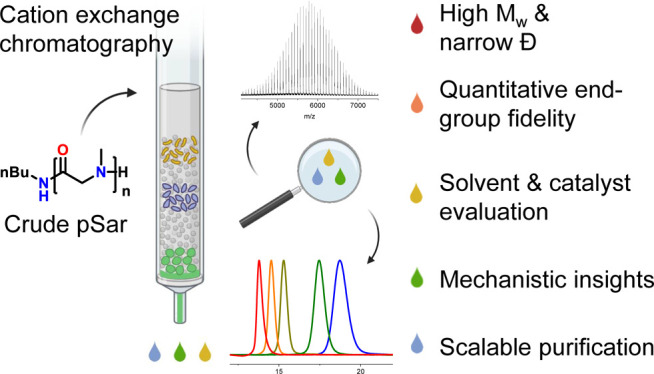

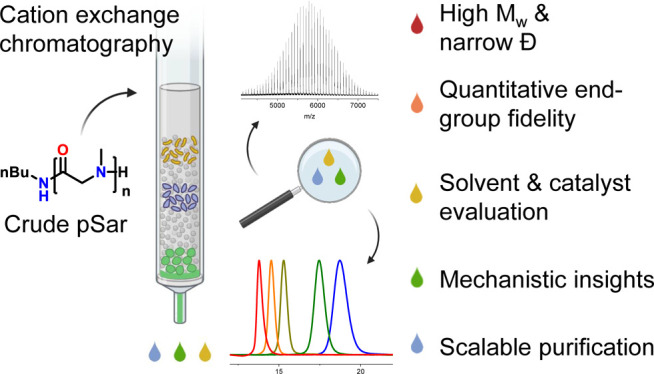

The conjugation of a water-soluble biocompatible polymer has presented itself as a simple solution to modulate the pharmacokinetic profiles of therapeutics such as small molecules, proteins, and nucleic acids, as well as potent drug delivery systems such as lipid-based nanoparticles or polymeric micelles.^1–5^ To date, poly­(ethylene glycol) (PEG) remains the most pharmaceutically relevant polymer applied in this strategy, prompting the popularization of the term PEGylation. Its success can be attributed to its high water solubility, good biocompatibility, and commercial availability from the laboratory to GMP grade. Additionally, the precise control over molecular weight, narrow molecular weight distribution, and high end-group fidelity guaranteed by the living anionic ring-opening polymerization^6^ and established production processes^7,8^ have fostered its overall success. Nevertheless, rising concerns about PEG-related immune responses and the rising prevalence of PEG-antibodies (accelerated blood clearance (ABC) phenomenon) in the general population^9–11^ have prompted research into PEG alternatives.^12,13^ Polysarcosine (pSar) is an emerging PEG alternative, featuring nearly identical solubility and chain rigidity in aqueous solutions,^14^ yet displaying attenuated immune responses.^14–18^

pSar is commonly synthesized through the ring-opening polymerization (ROP) of sarcosine N-carboxyanhydride (Sar-NCA), which belongs to the class of N-substituted NCAs (NNCA). NNCA ROPs generally exhibit a higher living character than NCA/N-thiocarboxyanhydride (NTA) ROPs, as the NNCA monomers lack a subtractable amide proton. Hence, propagation cannot proceed through an activated monomer mechanism (AMM), thus occurring exclusively by the normal amine mechanism (NAM). Similar to NCAs, NNCA polymerizations are sensitive to nucleophilic impurities and moisture, necessitating meticulous purification of monomers, solvents, and initiators to provide optimal control over the polymerization process. Consequently, research groups employ well-established solvent purification procedures and have explored different monomer synthesis and purification strategies. Despite advancements in purification,^19,20^ diminishing control over the molecular weight distribution is observed with increasing molecular weight (*M*_w_),^20^ suggesting that unidentified reactions compete with propagation, which compromises end-group fidelity.

Simultaneously, organocatalysis has emerged as a promising approach to improve control over the (N)­NCA ROPs, employing catalysts such as organic acids,^21–23^ trimethylsilane derivatives,^24^ tetramethylguanidine (TMG),^25,26^ crown ethers,^27,28^ and hydrogen-bond catalysts (e.g., Schreiner’s thiourea (sTU)).^29^ However, their impact on end-group fidelity remains unclear due to inherent characterization challenges associated with high *M*_w_ polymers via conventional techniques, such as MALDI-TOF-MS and NMR.^30,31^ Well-known shortcomings include the diminishing resolution as a function of *M*_w_ for MALDI-TOF-MS, while acquisition and processing challenges encumber quantitative end-group analysis via ^1^H NMR. To address these challenges in end-group analysis, we exploit the basicity of the pSar terminal amine in conjunction with ion exchange chromatography, to separate, identify, and quantify the different species as a function of synthetic parameters (Scheme 1). As a result, an automated scalable purification of pSar was established to routinely yield pSar with >97% purity across a wide range of molecular weights.

**Scheme 1:**
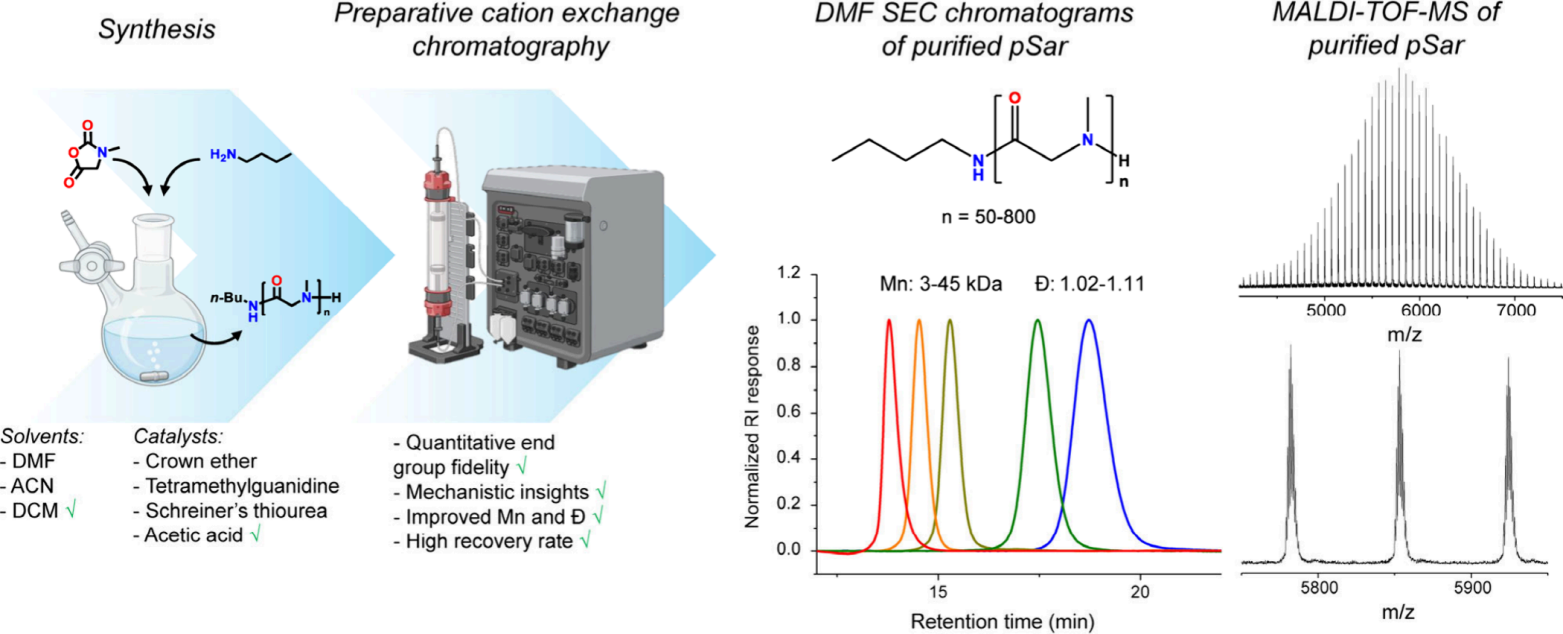
Schematic Overview of the Screening of pSar Polymerization Conditions and the Application of Cation Exchange Chromatography to (1) Isolate, Quantify, and Identify Impurities and (2) Obtain pSar with Quantitative End-Group Fidelity

Solvent effects: Initially, the impact of solvent on the end-group fidelity was examined as the polymerization of Sar-NCA has been reported in a wide range of solvents. Dimethylformamide (DMF) and dichloromethane (DCM) were selected as suitable solvents due to the compatibility of DMF with various polypept­(o)­ide structures,^32^ whereas DCM is reported to enhance propagation rates in NCA ROP. Finally, acetonitrile (ACN) was included due to its successful use in other ROPs, its dielectric constant value between DCM and DMF, and its ease of purification.^33^ With the purified solvents and Sar-NCA (Figures S1 and S2) in hand, we explored the polymerization of pSar with [M]/[I] ratios of 50–400 and examined their *M*_w_ distributions via size exclusion chromatography (SEC) in DMF (Figure 1Α–C) and the end-group fidelity via cation exchange chromatography (CEC) (Figure 1D–F). The latter technique separates mainly on electrostatic interactions of analytes with anionic ligands on the stationary phase.

**Figure 1:**
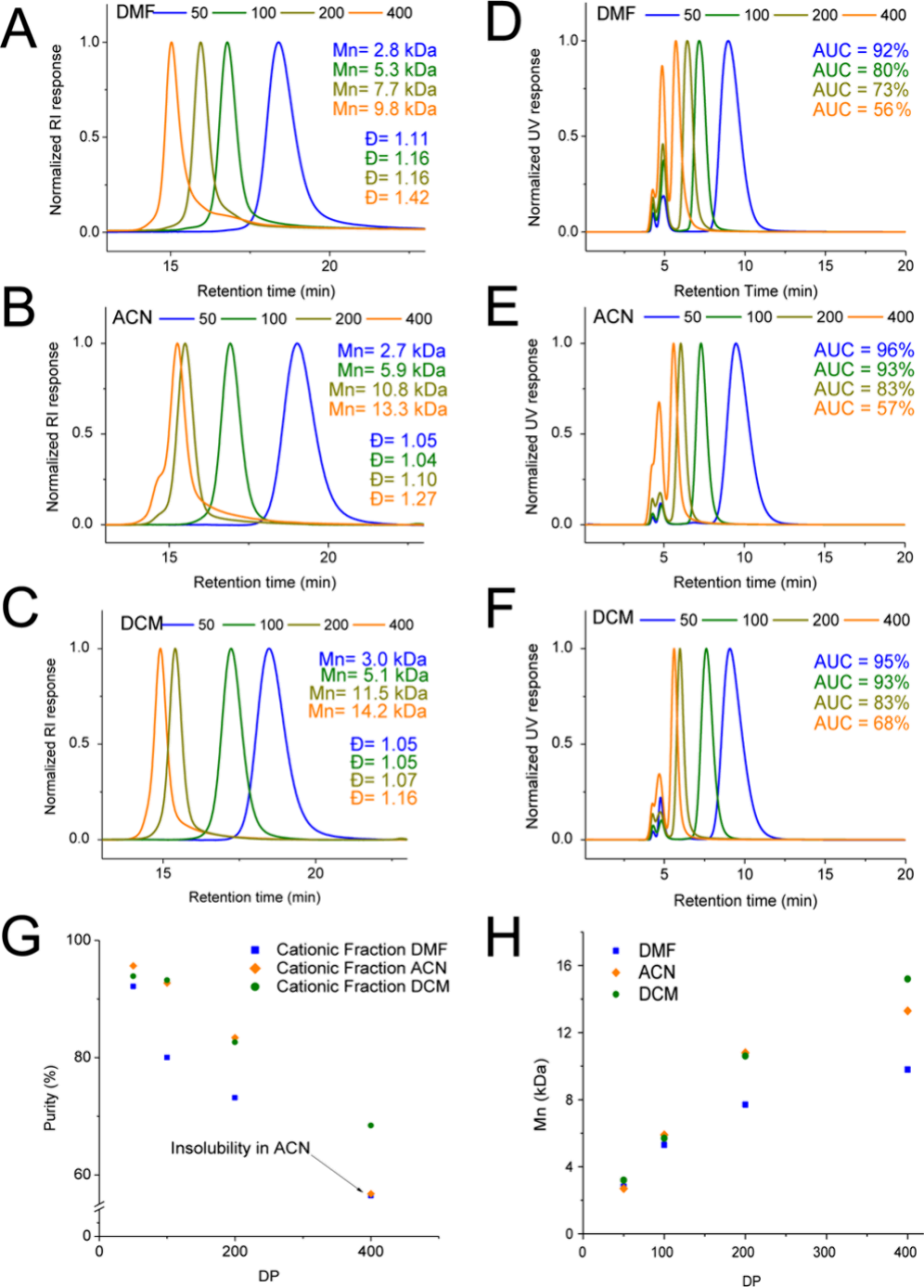
(A–C) DMF-SEC chromatograms of pSar with different [M]/[I] synthesized in DMF, ACN, and DCM, respectively. (D–F) CEC of pSar with different [M]/[I] synthesized in DMF, ACN, and DCM, respectively. (G) Purity as a function of DP of ROPs in different solvents as assessed by CEC. (H) *M*_n_ as a function of DP for ROPs in DMF, ACN, and DCM, respectively.

When examining the SEC plots across the different solvents, monomodal *M*_w_ distributions are obtained of relatively low dispersity (*Đ* < 1.2) up to DP200, whereas DP400 features *Đ* > 1.2, indicating a less-controlled ROP. pSar polymerized in DMF generally features lower *M*_n_ and higher *Đ* values (Figure 1A) compared to those polymerized in ACN or DCM. This can be attributed to DMF decomposition, generating species that interfere with the polymerization process.^34^ ACN and DCM demonstrate similar propagation rates and performance, yielding well-defined polymers for [M]/[I] ratios up to 400 in DCM and 200 in ACN (Figure 1B,C). ACN’s utility for [M]/[I] ratios over 200 is limited due to polymer insolubility at those DPs, thus compromising a controlled polymerization process.

Although monomodal *M*_w_ distributions are observed in SEC, CEC reveals three distinct signals, two of which have a constant retention time as a function of DP, indicating negligible interactions with the anionic resin. The third species shows a decreasing retention time as a function of DP, indicating the presence of a charged species that is increasingly shielded as a function of DP. (Figure 1D–F). An advantage of CEC is that its resolution can be adjusted by modulating the ionic strength and pH of the mobile phase (Figure S3). When the relative cationic content from the area under the curves (AUC), a gradual decline with an increase of *M*_w_ can be seen for all solvents (Figure 1G). Generally, products polymerized in DMF show the lowest cationic content, while DCM leads to improved end-group fidelity. The cationic content also correlates inversely with *Đ* obtained from SEC (Figure S4), proving that the sharp decline of the propagating amine end group compromises the living character of the ROP, inevitably resulting in a deviation of linearity of *M*_n_ as a function of DP (Figure 1H).

Organocatalysis: Although appropriate solvent selection can enhance the overall end-group fidelity, producing high-quality *M*_w_ polymers with high end-group fidelity remains challenging. To overcome these limitations, we investigated different organocatalysts (viz. 18-crown ether (CE), TMG, sTU, and acetic acid; Table S1) to energetically favor propagation over termination and chain transfer reactions, which was assessed for a [M]/[I] of 400.

Motivated by the application of CE to catalyze benzyl l-glutamate (BLG) NCA ROPs,^27,28^ we screened its application in Sar-NCA ROP. Unfortunately, CE provided no improvement over the noncatalyzed ROP, yielding a product with lower *M*_n_, comparable *Đ*, and reduced content of cationic end groups (Figure S5). Presumably, CE is not suited for NNCA ROP due to the lack of acidic protons in the NNCAs.

Next, we screened TMG catalysis, based on reports detailing its application in amine-initiated BLG-NCA ROPs.^25^ Unfortunately, the TMG-catalyzed product featured a considerably lower *M*_n_ compared to the noncatalyzed ROP, suggesting competitive initiation by both amine and TMG (Figure S6). Similar observations were made by Zhang et al. for TMG-mediated Sar-NTA polymerizations.^35^

Although sTU has been solely applied in NCA ROP in combination with various hydroxyl initiators, its catalytic activity was attributed to monomer activation and reversible protection of amine chain ends, besides increasing the nucleophilicity of the initiator,^29^ therefore presenting ample rationale for its exploration in the NNCA ROP of pSar. While sTU catalysis did increase the cationic content relative to the noncatalyzed ROP, both SEC and CEC revealed multimodal distributions, indicating the presence of additional low *M*_w_ species (Figure S7).

Lastly, we explored acetic acid-catalyzed ROP of NCAs, initially introduced by Bamford.^36^ It was successfully applied to Sar-NCA polymerization by Lu^23^ promoting propagation rates and improving control over the polymerization as a function of molecular weight. Encouraged by their work, we assessed the effect of AcOH as a catalyst on end-group fidelity of pSar across a DP range of 50 to 800. Our findings confirm the efficiency of acid catalysis, promoting fast and controlled polymerization of Sar-NCA and, more importantly, achieving higher cationic content compared to the noncatalyzed conditions. While this confirms a higher livingness of the ROP, a new peak was present in the CEC-trace, indicating the presence of side reactions, warranting further investigation (vide infra).

Although the investigated catalysts had a beneficial impact on chain propagation rates compared to noncatalyzed systems, CEC revealed that only AcOH improved the end-group fidelity of pSar and, consequently, the livingness of the ROP.

Mechanistic analysis: Due to the high purity of pSar of DP < 100, impurities in MALDI-TOF-MS spectra of crude products are easily overlooked, necessitating separation of the species. Encouraged by CEC results, we performed preparative purification of noncatalyzed and AcOH-catalyzed pSar polymers (Table S1). All isolated fractions for pSar50 and pSar100 were subsequently analyzed by MALDI-TOF-MS. The ROP in DCM contained a single nonionic fraction, containing a carbamic acid species in H- and Na- forms, which indicates that the rate-limiting decarboxylation of the carbamic acid species is slow^36–38^ relative to propagation, resulting in nonuniform chain growth and broadening of the *M*_w_ distribution. Additionally, a product of ω-end termination by an unidentified hydroxy-chloroalkyl chain with a bruto formula of C_6_H_12_OCl (Figure S8). This species likely originates from the amylene stabilizer in DCM, whereby radical DCM decomposition generates electrophilic amylene derivatives, which subsequently terminate the ROP (Figure S9).

For AcOH-catalyzed polymerizations, two separate noncharged fractions were collected (Figure 2). The first fraction contains the same chloroalkyl-terminated fragment as well as ω-acetyl pSar and zwitterionic α-COOH pSar. However, no fragments corresponding to carbamic acid could be identified, indicating that the addition of AcOH enhances the decarboxylation rate of carbamic acid relative to propagation. The second fraction contains a single fragment of a zwitterionic α-COOH terminal pSar. These findings confirm AcOH-mediated-initiation, presumably through the intermediate ammonium salt, which were recently reported as efficient initiators by Liu.^39^ The acetyl-terminal pSar confirms the presence of the anhydride initiating species, corroborating the observations of Ling for NNTA acid-catalyzed ROP.^22^ Notably, the zwitterionic fraction has a lower *M*_n_ distribution, indicating slow initiation by the carboxylate anion, while ω-acetyl and ω-chloroalkyl terminal species match with the intended *M*_n_ of pSar, suggesting that chain termination reactions are competing with propagation (Scheme 2). The cationic pSar fraction, however, is free of detectable side products and exclusively of heterotelechelic nature (Figure 3), making them ideal candidates for the synthesis of drug, protein, or lipid conjugates or block copolymers for the synthesis of polymer micelles (PM) or polyion complex micelles (PICMs).

**Figure 2:**
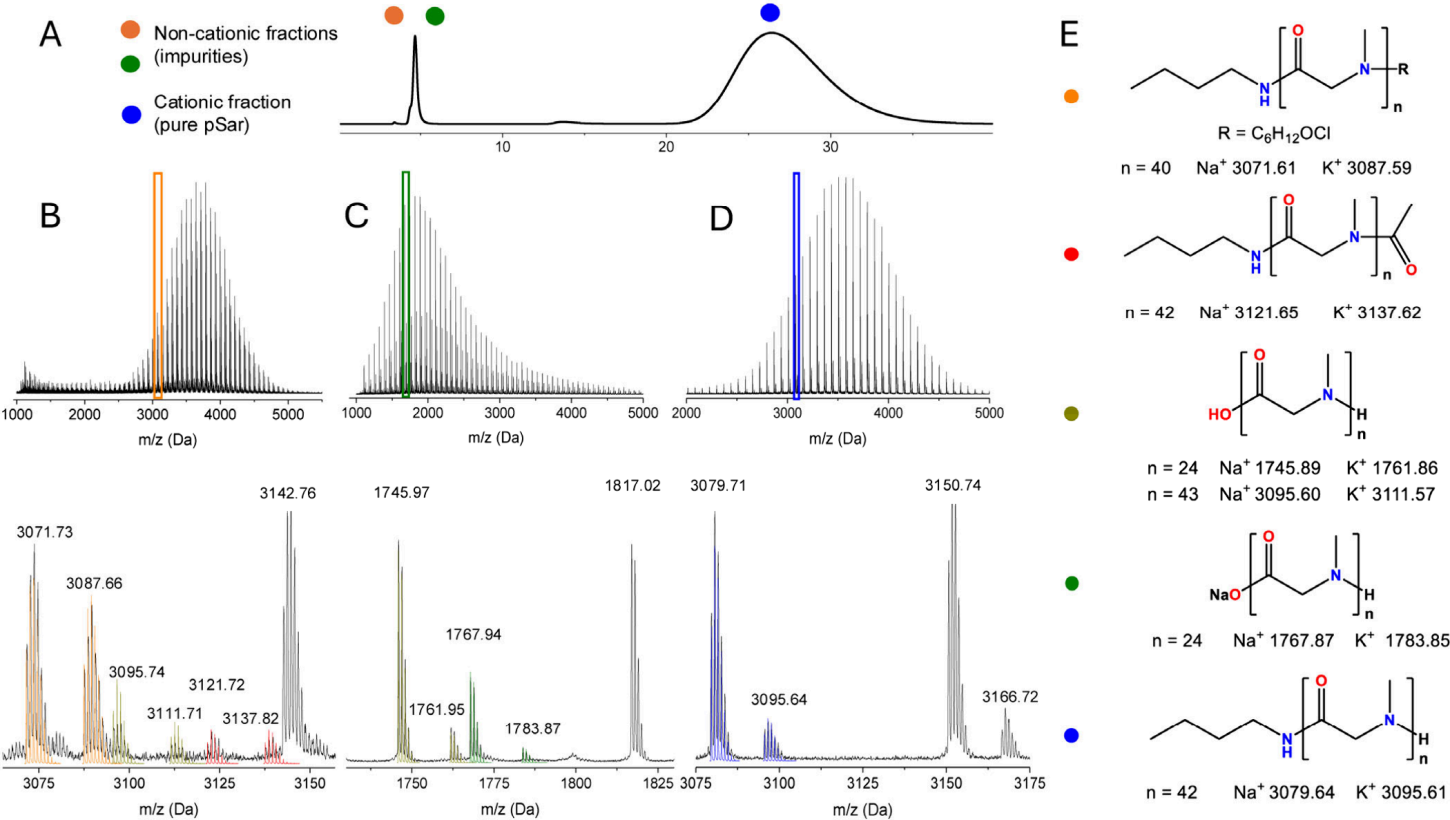
(A) CEC of pSar polymerized in DCM with AcOH as a catalyst, [M]/[I]/[A] = 50:1:5. (B–D) Full and zoomed MALDI-TOF-MS spectra of the respective fractions isolated by preparative cation exchange of acid-catalyzed pSar50. Experimental spectra are denoted in black and simulated in color, respectively. (E) Suggested species identified from MALDI-TOF-MS and their monoisotopic mass.

**Scheme 2:**
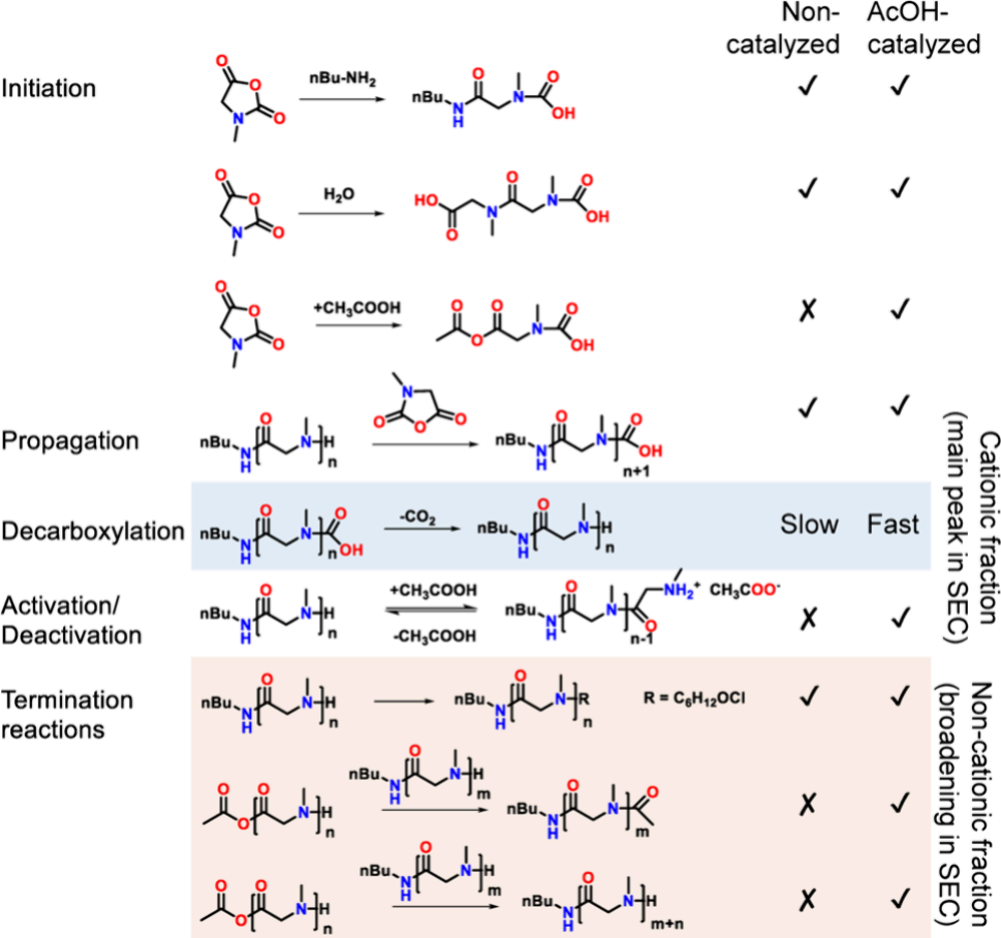
Suggested Pathway of Polymerization and Formation of Impurities During Non-catalyzed and Acid-Catalyzed Sar-NNCA ROP in DCM

**Figure 3:**
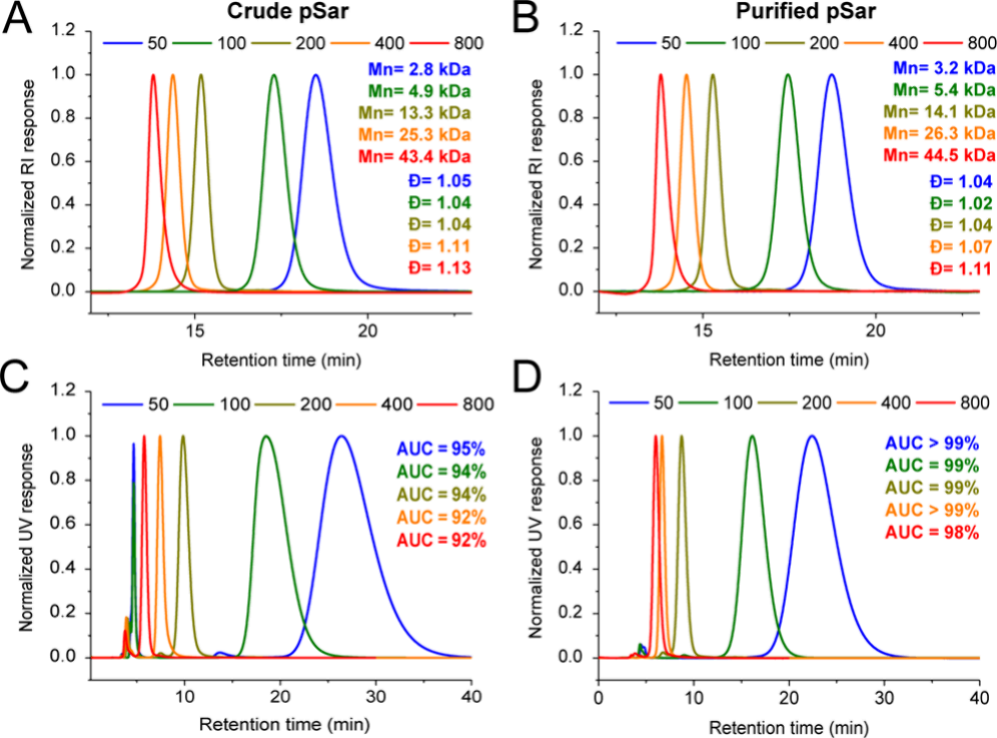
(A, B) DMF-SEC chromatograms of crude and purified by cation exchange pSar with different [M]/[I] synthesized in DCM with AcOH catalysis ([I]/[A] = 1:5). (C, D) CEC traces of crude and purified products.

In conclusion, we demonstrate the value of ion exchange chromatography on both analytical and preparative scales to monitor the end-group fidelity of pSar and separate species with different end groups, consequently granting mechanistic insights into the NNCA ring-opening polymerization and the full identification of byproducts. Our detailed analysis demonstrates that appropriate solvent selection can enhance the rate of the carbamic acid decarboxylation, yet contrary to recent literature,^40^ solvents alone cannot overcome this critical bottleneck. To overcome this limitation, we explored a variety of organocatalysts that have been showcased in contemporary research to accelerate the NCA ROP. However, our results reveal that their effects on polypeptoid end-group fidelity, and consequently product quality, are more nuanced. Of the investigated catalysts, only organic acids significantly improve ω-end-group fidelity by (1) enhancing the relative rate of decarboxylation to propagation and (2) establishing a rapid exchange of dormant ammonium carboxylates and propagating free amines. Yet, the introduction of AcOH also generates additional side products. Further purification of acid-catalyzed products via preparative ion exchange presents a scalable method toward well-defined heterotelechelic pSar across a wide range of DPs, which we demonstrated for DP 50–800 (Figure S10). This approach can be extended to other NNCAs and facilitate the development of polypept­(o)­ides as therapeutics, addressing manufacturing challenges to comply with the stringent regulatory standards in pharmaceutical applications.

## Supplementary Material



## Notes

Experimental details, comprehensive characterization data, and explanatory notes (PDF)
