# Supplementary material for: Toward Quantitative End-Group Fidelity in the Synthesis of High Molecular Weight Polysarcosine
Source: ACS Macro Lett. 2025 Apr 15;14(5):532–7. doi: 10.1021/acsmacrolett.5c00165 (PMC12096423; doi:10.1021/acsmacrolett.5c00165)
Supplement: Supplementary file 1 [file mz5c00165_si_001.pdf]

SUPPORTING INFORMATION FOR:

Towards Quantitative End-Group Fidelity in the Synthesis of High Molecular Weight Polysarcosine

Zlata Nagorna[1], Matthias Barz[1,*], Joachim F. R. Van Guyse[1,*]

[1]Leiden Academic Centre for Drug Research (LACDR), Leiden University, Einsteinweg 55, 2333 CC Leiden, The Netherlands

*Joachim F. R. Van Guyse Email: j.f.r.van.guyse@lacdr.leidenuniv.nl
*Matthias Barz Email:m.barz@lacdr.leidenuniv.nl

Table of contents

1. **Materials**
2. **Methods**
3. **Sarcosine-NNCA (Sar-NNCA) synthesis**
4. **Representative polymerization procedure of Sar-NCA**
5. **Representative polymerization procedure of Sar-NCA with the addition of a catalyst**
6. **Increasing resolution of analytical cation exchange for high Mw polymers**
7. **Summary of polymerization conditions and characterization data for crude and purified by cation exchange chromatography pSar.**
8. **Additional characterization data**
9. **Automated preparative cation exchange chromatography and scalability**
10. **References**

1. Materials

All chemicals were used as received unless specified otherwise.

DMF for GPC (VWR, CHROMANORM >99.9%), ACN (ROTH, ROTISOLV >99.9%, DNA synthesis grade), DCM (Honeywell, >99.9%), THF (VWR, Normapur, >99.8%), Diethyl ether (Honeywell, >99.5%), Benzene (TCI, >99.5%), CaH2 (Thermo Scientific, ca. 93%),p-Toluenesulfonyl isocyanate (Sigma Aldrich, 96%), Butylamine (TCI, >99%), Sarcosine (Thermo Scientific, 98%), Triphosgene (Fluorochem), Acetic acid (Sigma Aldrich, 99-100%), 18-Crown ether (Sigma Aldrich, >99%), 1,3-Bis[3,5-bis(trifluoromethyl)phenyl]thiourea, (BLDPharm, 98%), Tetramethylguanidine (Sigma Aldrich, 99%), LiCl (VWR Life Science, biotechnology grade), Sodium phosphate monobasic dihydrate (Fluka analytical, di-Sodium hydrogen phosphate dodecahydrate (Roth, >99%), α-cyano-4-hydroxycinnamic acid (Sigma Aldrich, >98%), Sodium Trifluoroacetate (Sigma Aldrich, 98%), Barium Oxide (Chem Cruz, > 90%).

DMF was distilled over BaO and ninhydrin at 20 mbar and stored under an inert atmosphere protected from the light. Acetonitrile was refluxed for 2 hours over p-toluenesulfonyl isocyanate (1 mL/100 ml of acetonitrile) under an inert atmosphere to remove any nucleophilic impurities prior to fractional distillation. The purity was confirmed by the absence of color after adding 5 µL of Sanger's reagent to a 1 mL aliquot. DCM was stirred overnight with CaH_2 prior to fractional distillation under an inert atmosphere. Dry THF was obtained from a PureSolve Microsystem (Inert Corporation).

2. Methods

Size-exclusion chromatography (SEC) was measured on a Jasco HPLC system equipped with an inline degasser, a photodiode array detector (MD-4010), a refractive index detector (RI-4030) and a column oven (CO-4060) set to 40 °C equipped with G4000HHR and G3000HHR (TOSOH, Japan) columns and a guard column in series. The mobile phase was DMF with 10 mM of LiCl at a flow rate of 0.8 mL/min. The number average molecular weight (Mn) and dispersity (Đ) were calculated against polyethylene glycol (PEG) standards obtained from Agilent (Mp range = 99kDa - 600 Da).

Analytical cation exchange chromatography (analytical CAIEX) was measured on a Waters HPLC system equipped with Waters 515 HPLC pump, Gilson 234 autosampler, Waters 2998 PDA detector, Waters 2410 RI detector, and a TSKgel SP-5PW column (TOSOH, Japan). The mobile phase was a 2 mM or 0.5 mM phosphate buffer (pH 6.5) at a flow rate of 0.5 mL/min.

Fourier-transform infrared spectrometry (FT-IR) was recorded on an FT/IR-4100 (JASCO) fitted with an ATR accessory (MIRacleTM, Pike Technologies). Polymerization progress was tracked via the decrease of characteristic monomer IR bands at 1853 and 1786 cm^{-1}.

NMR spectra were recorded using a Bruker Avance 400 MHz Wide Bore NMR spectrometer (Bruker), and the 1H chemical shifts are reported as parts per million (ppm) relative to tetramethylsilane using $CDCl_3$ as solvent. Raw NMR data was processed using Mestrenova 12.0.0 software package.

MALDI-TOF-MS measurements were performed on a Sciex 4800 MALDITOF/TOF MS instrument equipped with an Nd:YAG laser (200 Hz, 355 nm) controlled by 4000 Series Explorer software version 3.5.3 (Applied Biosystems, Germany). The instrument was operated in positive ion mode with delayed extraction and an acceleration voltage of 20 kV with a grid of 15.6 kV. Analyte solutions were

prepared by mixing 10 µL of a 20 mg/mL solution of α-cyano-4-hydroxycinnamic acid (Sigma Aldrich) with 4 µL of a 4 mg/mL polymer solution and 2 µL of a 2 mg/mL solution of sodium trifluoroacetate (Sigma Aldrich). Generally, MeOH was utilized as the solvent. The analyte solutions were subsequently spotted on a stainless steel 96-well sample plate according to the dried droplet method. The same well was spotted at least twice with the analyte solution. Spectra were acquired in positive ion reflector mode, with a 20 kV acceleration voltage and delayed extraction, using the built-in software. Spectra were acquired with at least 500 shots per measurement, while reflector voltage was kept as low as possible for each sample. Raw data was processed using mMass 5.5.0 software.

Preparative cation exchange chromatography was performed using Toyopearl SP650M resin. Resins were packed in-house into empty cartridges from Biotage for use on NGC Discovery 10 system equipped with a multi UV-Vis/conductivity detector, pH probe, and BioFrac fraction collector, allowing the automation of the process. Columns were washed with 3-4 column volumes of 50 mM HCl followed by MQ water until the pH exceeded 6. Crude pSar samples were dissolved in a small amount of MQ water and loaded on the column with an ASX-560 Teledyne autosampler. Elution was performed using a step gradient of MQ water followed by a switch to 50-100 mM ammonia solution. The elution of the polymer was tracked via UV signal at 220 nm. The solvents were removed in vacuo, and fractions were freeze-dried.

3. Sarcosine-NNCA (Sar-NNCA) synthesis

Sarcosine (20 g, 224.5 mmol, 1 eq) was weighed and ground into a fine powder, placed into a pre-dried two-neck round-bottomed flask, and dried from benzene in vacuo overnight. Dry THF (250 mL) was added under a steady nitrogen flow, followed by triphosgene (33.3 g, 112 mmol, 0.5 eq). The colorless suspension was heated to 70 °C, turning into a dark orange clear solution after 2 hours of stirring. A steady flow of dry nitrogen was led through the solution into two gas washing bottles filled with aqueous sodium hydroxide solution to remove excess HCl and phosgene for 2-3 hours. This led to partial evaporation of THF and the formation of an off-white precipitate, which was collected by filtration under an inert atmosphere. The filtrate was subsequently washed with 100 mL dry THF. The crude product was sublimed at 80 – 85 °C and 0.6-0.9 mbar. The final product was collected from the sublimation apparatus under an inert atmosphere. Yield = 8.747 g (34%).

^{1}H NMR (400 MHz, $CDCl_3$) δ 4.16 (s, 1H), 3.09 (s, 2H).

^{13}C NMR (101 MHz, $CDCl_3$) δ 165.41, 152.43, 51.03, 30.42.

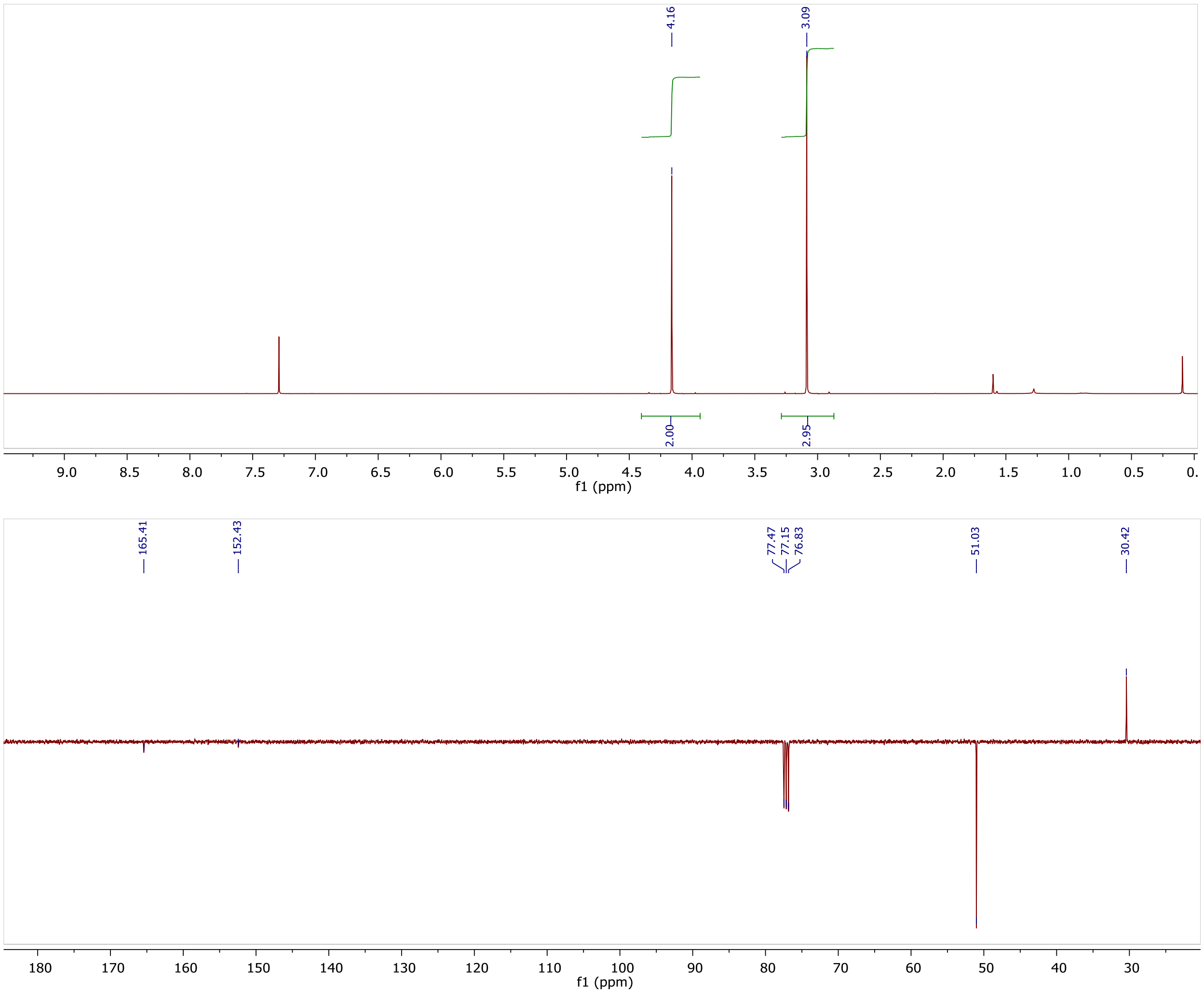

Figure S1. ^{1}H NMR (top) and ^{13}C-APT NMR (bottom) of a saturated solution of sublimed Sar-NNCA.

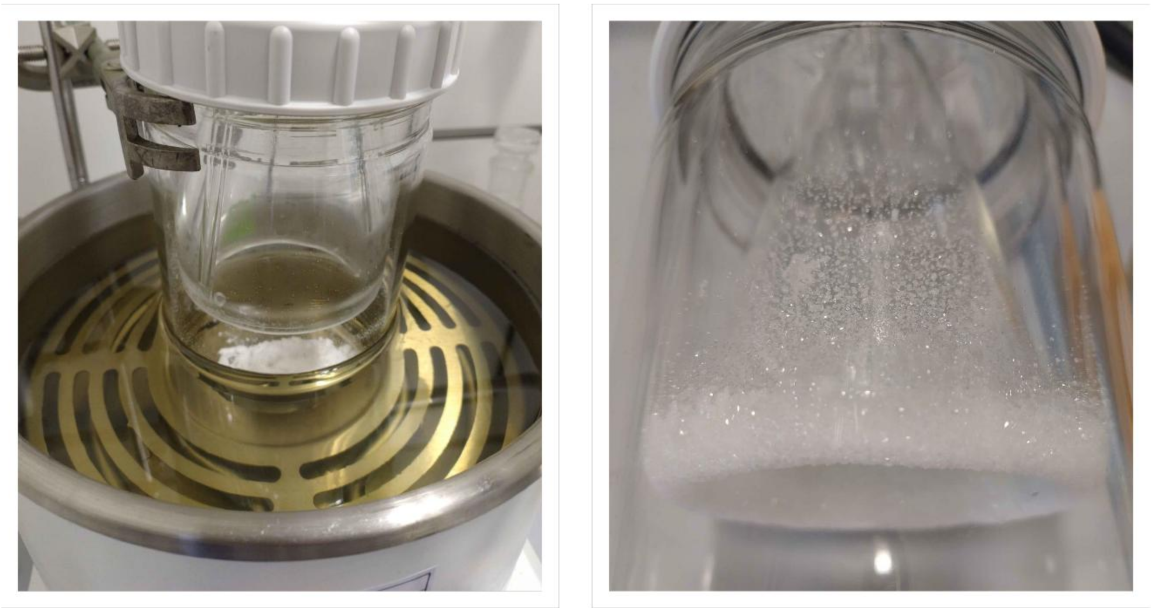

Figure S2. Sarcosine NNCA before (left) and after (right) sublimation.

4. Representative polymerization procedure of Sar-NCA

Sar NNCA (50-400 eq) was weighted into a vacuum-dried Schlenk flask and dissolved in an adequate volume of dry DCM to reach a 0.4 M monomer concentration. After that, butylamine (1 eq) was added to the polymerization mixture under N_2 flow. The Schlenk flask was closed with a rubber septum, and a balloon filled with N_2 was added. The polymerization was left to stir at room temperature, and the monomer conversion was monitored by FT-IR. Further, the polymerization mixture was precipitated in a tenfold excess of cold ether, centrifuged, and lyophilized.

5. Representative polymerization procedure of Sar-NCA with the addition of a catalyst

Sar NNCA (50-800 eq) was weighted into a vacuum-dried Schlenk flask and dissolved in an adequate volume of dry DCM to reach a 0.4 M monomer concentration. After that, a catalyst (AcOH, CE, sTU or TMG) (1 or 5 eq) was added, followed by butylamine (1 eq), to the polymerization mixture under N_2 flow. The Schlenk flask was closed with a rubber septum, and a balloon filled with N_2 was added. All catalysts were used as stock solutions in ACN at high concentrations to ensure precise addition, due to the volatility and high density of DCM. The polymerization was left to stir at room temperature, and the monomer conversion was monitored by FT-IR. Finally, the polymerization mixture was precipitated in a tenfold excess of cold ether, centrifuged, and lyophilized.

6. Increasing resolution of analytical cation exchange for high Mw polymers

Cation-exchange resins from TOSOH are based on basic size exclusion beads HW-65 (hydroxylated methacrylate beads), functionalized with different anionic ligands, hence, they display dual separation properties based on both charge and hydrodynamic volume. Typically, non-charged species display little to no interactions with the column material, and their retention time is only influenced by column parameters and flow rate, while the retention time of charged species is also influenced by pH and salt concentration of the mobile phase. For our analytical cation exchange system equipped with a TSKgel-SP-5PW (7.5mm diameter) column, the typical elution time of non-charged species varied from 3 to 5 minutes, and charged species from 5 to 45 min, depending on the mobile phase used. We observed that for pSar with chain lengths above 200, the cationic and non-cationic species partially coelute in 2 mM phosphate buffer (pH 6.5) as a mobile phase, making quantification difficult (Figure S3B). It is possible to improve the resolution by decreasing salt concentration in the eluent, which we successfully achieved by switching to 0.5 mM phosphate buffer (Figure S3 A). Deviations in retention time are possible due to the high sensitivity of the method to salt concentration and pH, especially in low concentration salt buffers.

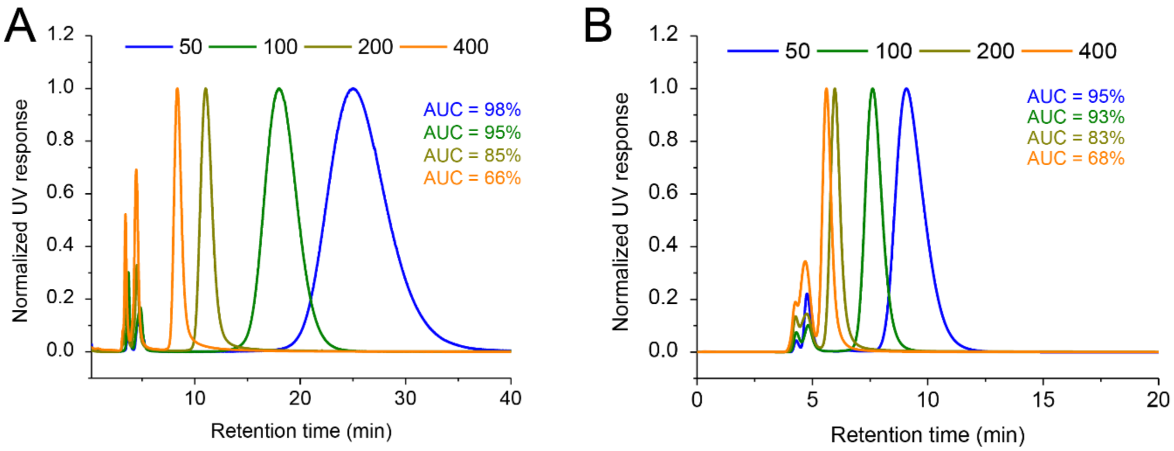

Figure S3. Analytical cation exchange chromatograms of pSar in DCM measured with 0.5 mM (A) and 2 mM PB (B) buffer (pH 6.5) as mobile phase, respectively.

7. Summary of polymerization conditions and characterization data for crude and purified by cation exchange chromatography pSar.

Table S1. Summary of polymerization and intermediate purification of pSar by cation exchange chromatography.

Conditions	[M]/[I]/[Cat]	$[M]_0$ (mol/L)	Crude pSar			Purified pSar		
			Mn (kDa)	Đ	AUC (%)	Mn (kDa)	Đ	AUC (%)
DCM	50/1/0	0.4	2.8	1.09	94	2.8	1.07	>99
	50/1/0	0.4	3.2	1.05	98	3.3	1.04	>99
	100/1/0	0.4	5.0	1.05	93	5.0	1.04	>99
	100/1/0	0.4	5.7	1.04	95	5.7	1.02	>99
	200/1/0	0.4	11.5	1.07	83	12.1	1.03	97
	200/1/0	0.4	10.6	1.07	85	-	-	-
	400/1/0	0.4	14.2	1.16	68	15.8	1.06	97
	400/1/0	0.4	15.2	1.23	66	-	-	-
	100/1/0	1	10.3	1.03	75	-	-	-
	400/1/0	1	18.7	1.34	57	-	-	-
DCM/CE	400/1/1	0.4	12.7	1.3	53	-	-	-
DCM/TMG	400/1/1	0.4	11.0	1.13	72*	-	-	-
DCM/TU-S	400/1/1	0.4	17.5	1.1	75*	-	-	-
DCM/AcOH	50/1/5	0.4	2.8	1.05	95	3.2	1.04	>99
	100/1/5	0.4	4.9	1.04	94	5.4	1.02	99
	200/1/5	0.4	13.3	1.04	94	14.1	1.04	99
	400/1/5	0.4	25.4	1.11	92	26.3	1.05	>99
	400/1/1	0.4	24.5	1.08	91	-	-	-
	800/1/5	0.4	43.4	1.13	92	44.5	1.11	98

AUC (%) represents the area under the curve of a cationic peak in SEC. *multimodal peaks in SEC.

8. Additional characterization data

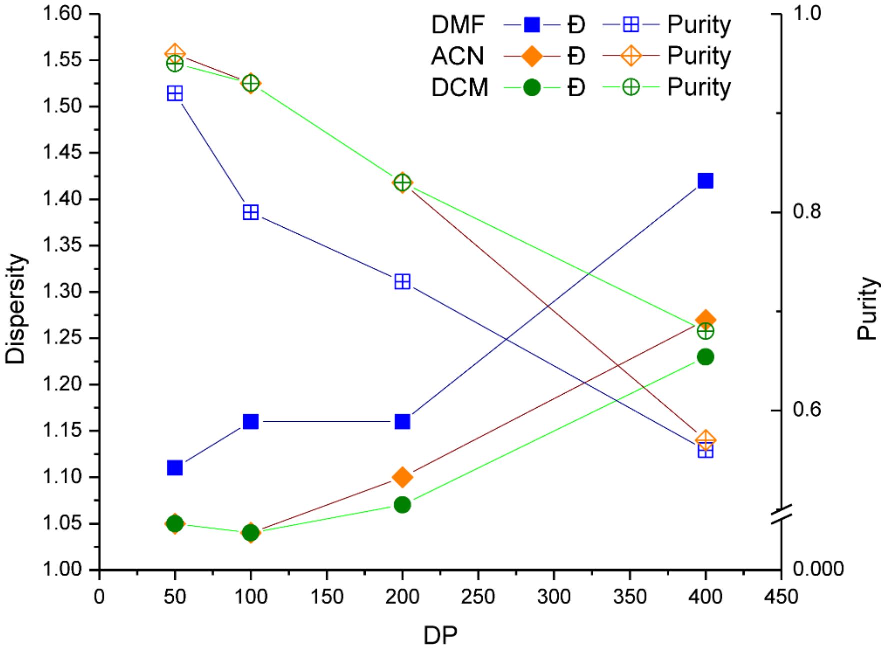

Figure S4. Relationship between dispersity values and purity (cationic content) in crude pSar samples polymerized in DMF/ACN/DCM.

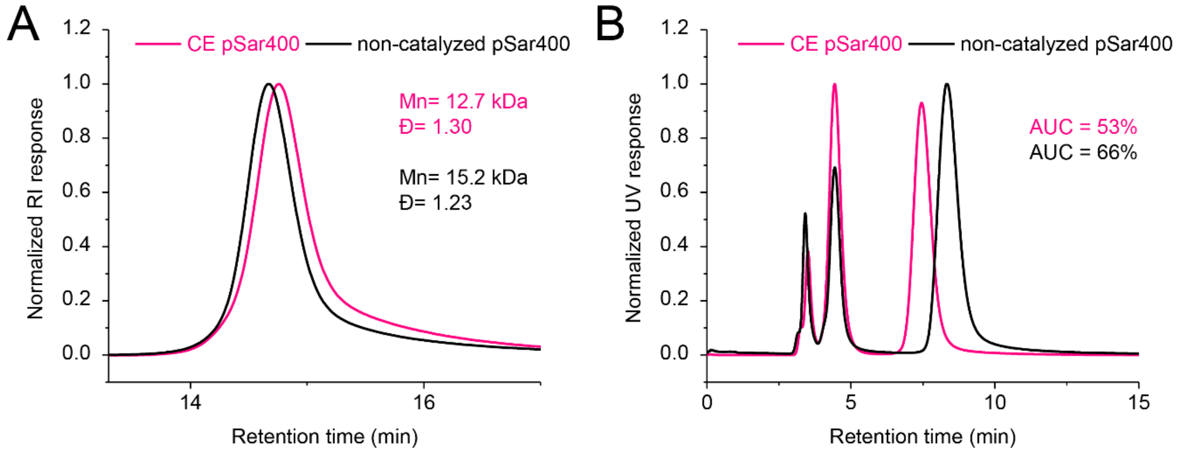

Figure S5. A) DMF SEC chromatogram of 18-crown-ether (CE) catalyzed and non-catalyzed pSar polymerized in DCM with [M]/[I] 400:1. B) Analytical cation exchange of CE-catalyzed and non-catalyzed pSar, respectively.

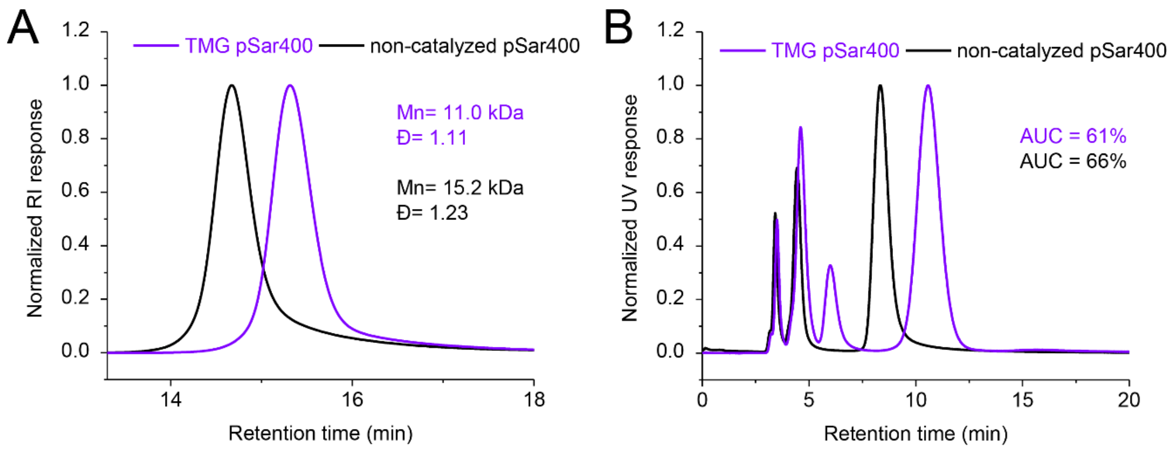

Figure S6. A) DMF SEC chromatogram of tetramethylguanidine (TMG) catalyzed and non-catalyzed pSar polymerized in DCM with [M]/[I] 400:1. B) Analytical cation exchange of TMG-catalyzed and non-catalyzed pSar, respectively.

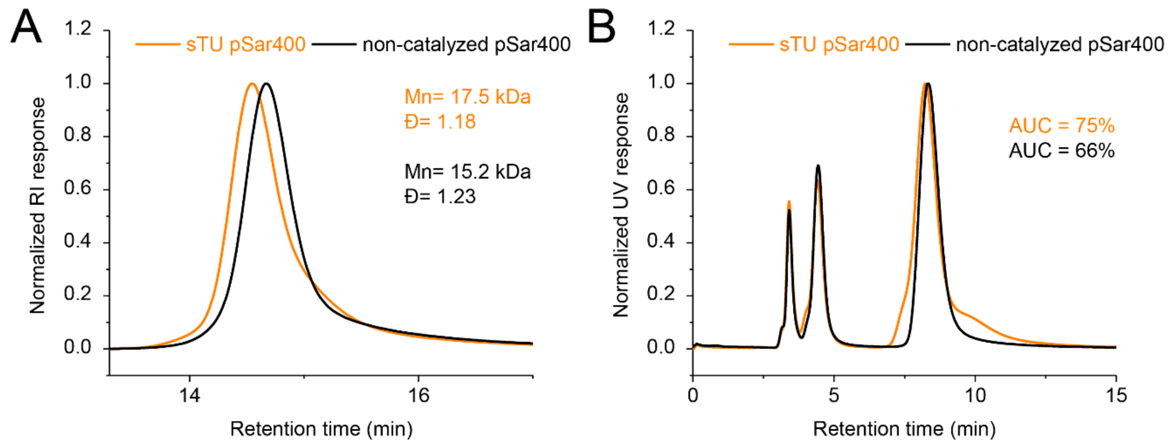

Figure S7. A) DMF SEC chromatogram of Schreiner's thiourea (sTU) catalyzed and non-catalyzed pSar polymerized in DCM with [M]/[I] 400:1. B) Analytical cation exchange of sTU-catalyzed and non-catalyzed pSar, respectively.

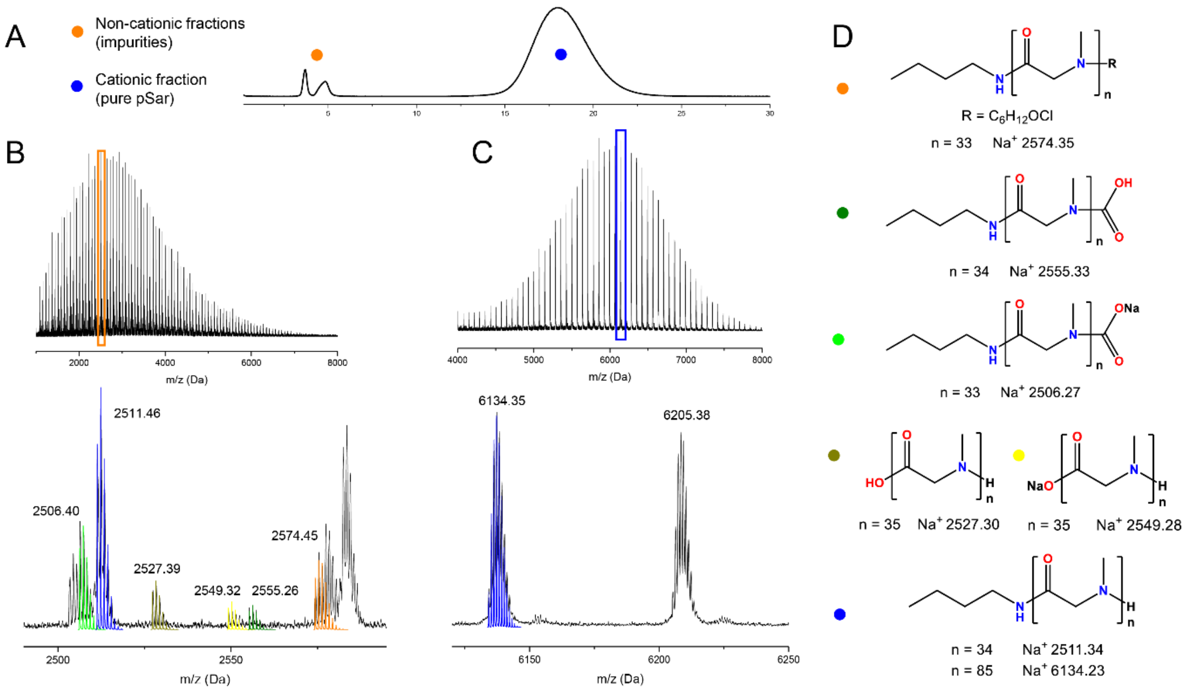

Figure S8. A) Cation exchange chromatogram of pSar polymerized in DCM [M]/[I]= 100:1 B, C) Full and zoomed MALDI-TOF-MS spectra of the respective fractions isolated by preparative cation exchange of pSar100. Experimental spectra are denoted in black, and simulated peaks in color, respectively. D) Suggested structures of fragments identified from MALDI-TOF-MS and their monoisotopic mass.

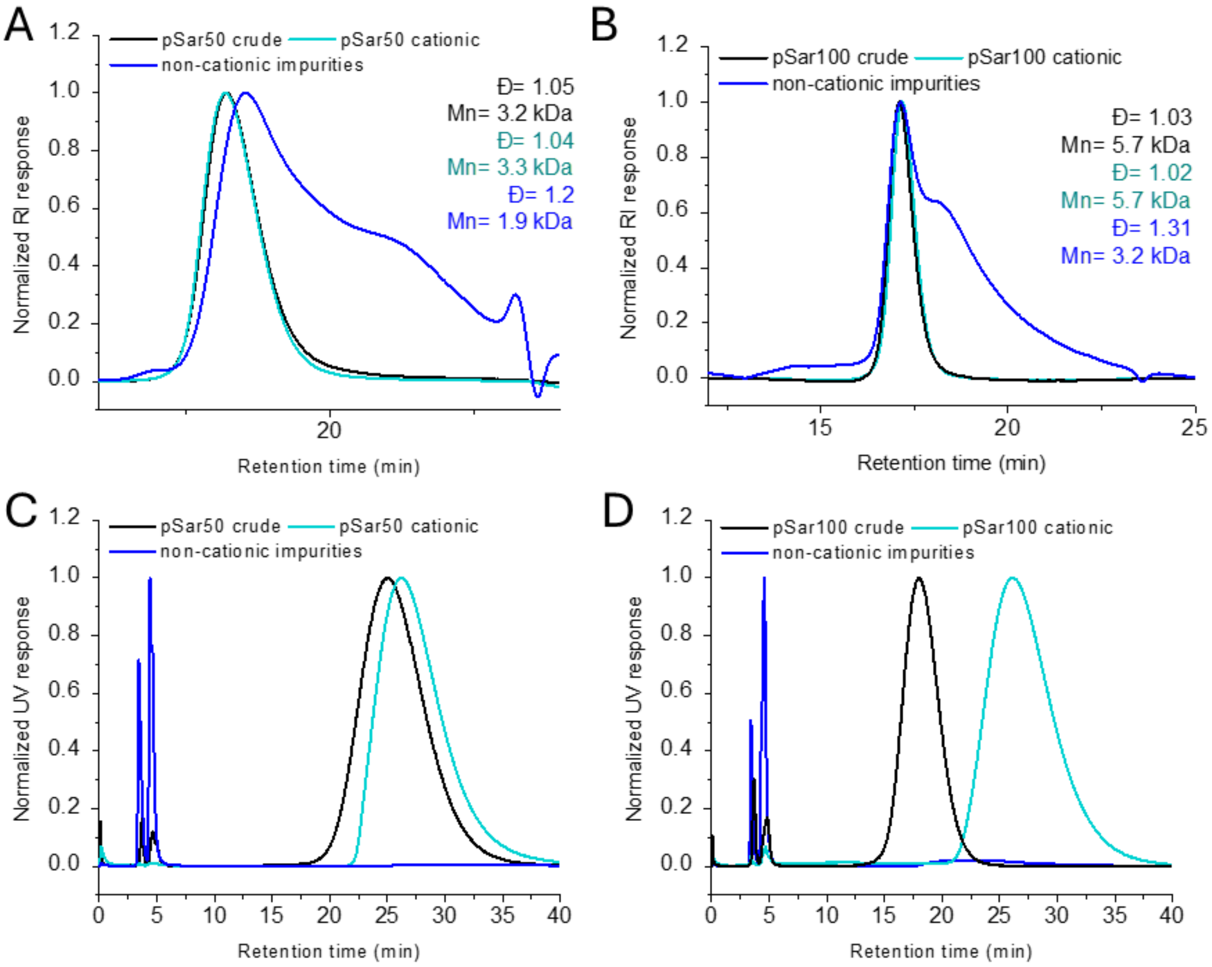

Figure S9. A,B) DMF SEC chromatograms of crude material, cationic and non-cationic fractions of non-catalyzed pSar50 and pSar100 polymerized in DCM, respectively. C) Analytical cation exchange chromatogram of crude material, cationic and non-cationic fractions of non-catalyzed pSar50 polymerized in DCM. The mobile phase was 0.5 mM PB (pH 6.5) for all samples. D) Analytical cation exchange chromatogram of crude material, cationic and non-cationic fractions of non-catalyzed pSar100 polymerized in DCM. The mobile phase was 0.5 mM PB (pH 6.5) for cationic and non-cationic fractions and 2 mM PB (pH 6.5) for the crude material.

9. Automated preparative cation exchange chromatography and scalability

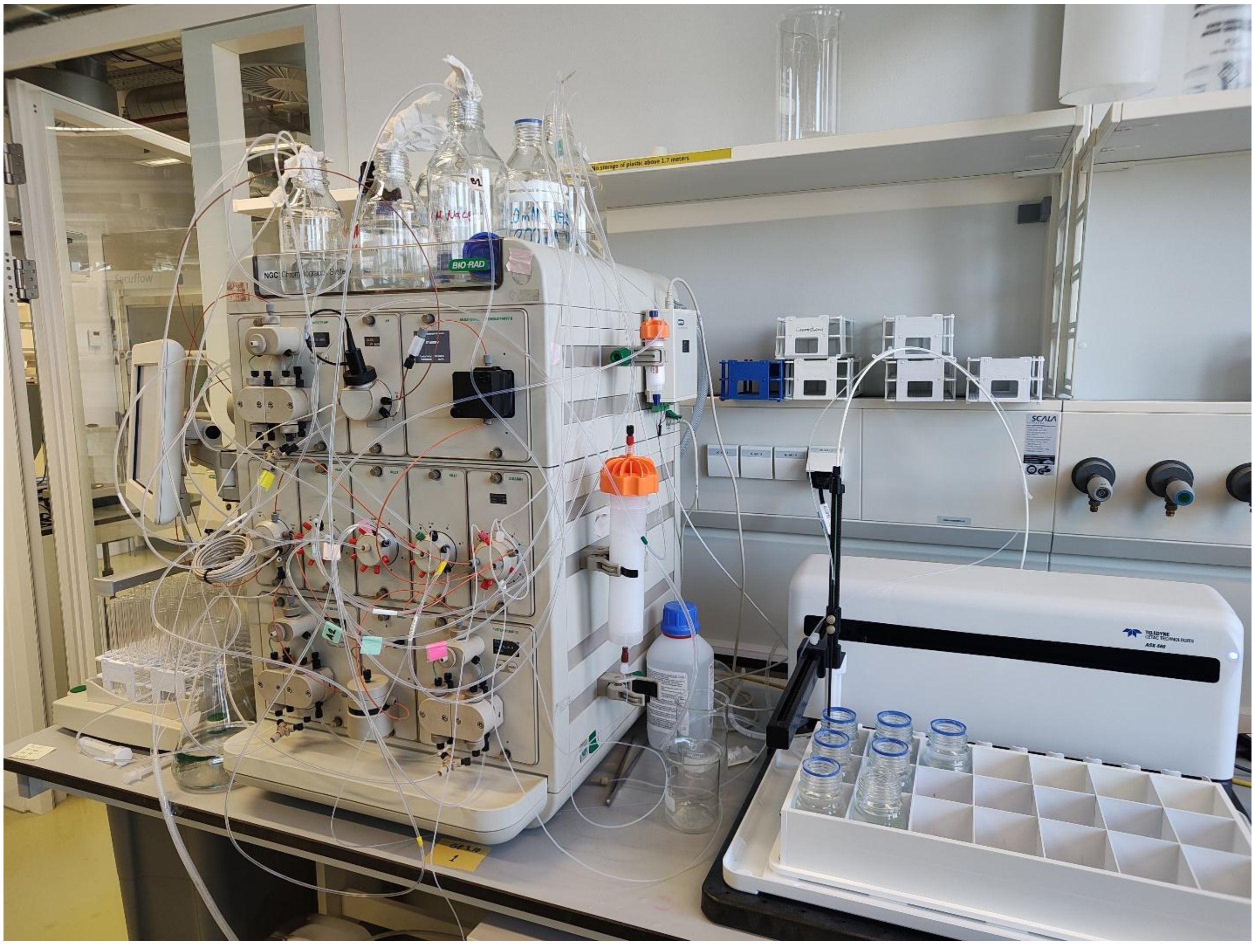

Figure S10. BIO-RAD NGC Discovery 10 system equipped with an ASX-560 Teledyne autosampler utilized for the preparative purification of pSar on multigram scale.

Scalability: The scalability of ion-exchange chromatography is evident from the pharmaceutical industry, whereby ion exchange chromatography is performed on large scale (from gram to kilogram scale) to purify peptides, proteins[1] and nucleic acids,[2] such anti-sense oligonucleotides and siRNA.

10. References

1. Mollerup, J. M., Hansen, T. B., Kidal, S., Sejergaard, L. & Staby, A. Development, modelling, optimisation and scale-up of chromatographic purification of a therapeutic protein. *Fluid Phase Equilib* 261, 133–139 (2007).
2. Kobl, K. *et al.* Oligonucleotide Purification by Ion Exchange Chromatography: A Step-by-Step Guide to Process Understanding, Modeling, and Simulation. *Org Process Res Dev* 28, 2569–2589 (2024).
